# In vitro and in vivo hepatotoxicity study of Afriplex™ GRT through an inflammatory response

**DOI:** 10.1016/j.toxrep.2022.10.006

**Published:** 2022-10-17

**Authors:** Kwazikwakhe B. Gabuza, Ntandoyenkosi Buthelezi, Abidemi Paul Kappo, Thendo I. Mabuda, Rebamang Mosa, Johan Louw, Christo J.F. Muller

**Affiliations:** a Biomedical Research and Innovation Platform, South African Medical Research Council, Cape Town, South Africa; b Centre for Cardio-metabolic Research in Africa (CARMA), Division of Medical Physiology, Faculty of Medicine and Health Sciences, Stellenbosch University, Cape Town, South Africa; c Department of Biochemistry and Microbiology, University of Zululand, eMpangeni, South Africa; d Department of Biochemistry, Genetics and Microbiology, University of Pretoria, Tshwane, South Africa; e Department of Biochemistry, University of Johannesburg, Johannesburg, South Africa; f DSI/Mintek Nanotechnology Innovation Centre, Department of Biotechnology, University of the Western Cape, Cape Town, South Africa

**Keywords:** Rooibos, Aspalathin-rich, Sprague Dawley, HepG2/C3A, Hepatotoxicity

## Abstract

**Background:**

The focus on traditional and complementary medicine for supplementation and treatment of diseases is high. Aspalathus linearis commonly known as Rooibos showed several beneficial effects, this led to the standardized production of a pharmaceutical grade green rooibos extract (Afriplex TM GRT) with enhanced polyphenolic content. The aim of this study was to assess toxicity of Afriplex TM GRT in HepG2/C3A cells and Sprague Dawley rats.

**Methods:**

Afriplex GRT TM (0.1, 1, 10, 100, or 1000 μg/mL) in DMSO was added to the media to the final 0.01% DMSO for treatment of HepG2/C3A for 1, 24 and 48 hrs followed by MTT and ATP assays. Sprague Dawley rats were grouped to Control, Afriplex TM GRT treated (10, 100 and 300 mg/kg); and acute (24hrs tetrachloromethane (CCl 4) injected hepatotoxicity control). Serum biochemistry, histology and Western blot analysis on liver were performed.

**Results:**

Afriplex TM GRT significantly reduced cell viability at 100 and 1000μg/mL after 48 hrs. Acute CCl 4 treatment significantly increased serum alanine aminotransferase in rats. The highest extract treatment of 300 mg/kg significantly elevated aspartate amino transferase. There was severe macro vesicular in the CCl 4 group whereas mild to moderate micro vesicular steatosis was seen in the 300 mg/kg Afriplex TM GRT treated group. Highest extract treatment significantly reduced NFkB expression on Western blot analysis.

**Conclusion:**

The beneficial effects of pharmaceutical grade Afriplex GRT TM are concentration and dosage based. Afriplex GRT TM exerts its beneficial effects via NFkB as demonstrated by the dose dependent reduction of NFkB on Western blot analysis. More work need to be done to explore the exact mechanism that occurs in the NFkB pathway.

## Highlights

• Afriplex GRT sub-chronic administration to Sprague Dawley does not impact the liver histology.
• High dosage of Afriplex GRT increases the AST levels within acceptable ranges.
• Inflammatory genes expression showed no response to high levels of Afriplex GRT™
• Toxicity of plant based active medicinal products need to be tested for toxicity.
• Sprague Dawley responded to acute carbon tetrachloride with macro vesicular steatosis.

## Graphical Abstract

**Figure:**
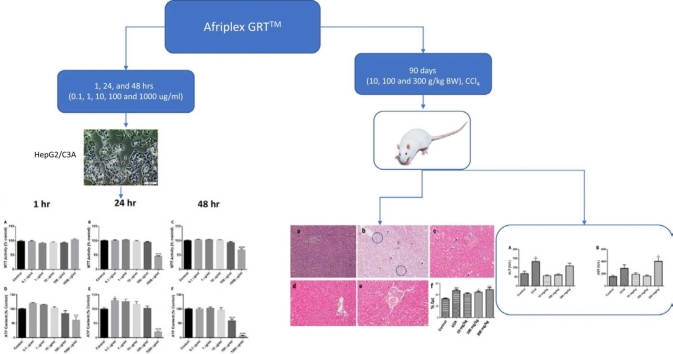

## 1 Introduction

The liver is the body’s engine, it is responsible for energy homeostasis, protein synthesis, detoxification, and metabolism of drugs and other xenobiotics. However, amid performing these processes, the liver gets predisposed to dangerous toxins which lead to liver damage or injury. Drug-induced liver injury (DILI) is the most common cause of hepatotoxicity affecting both young and old age groups. From a prospective population-based French study with an annual estimated incidence of 13.9 + /- 2.4 DILI cases per 100,000 inhabitants, it has been extrapolated that nearly 44,000 individuals in the United States will suffer from DILI each year [1]. DILI is not having a global statistic, with South Africa having the statistic that is focused on HIV and TB DILI. Hepatotoxicity is the resultant effect of the accumulation of harmful chemicals or toxins in the system that exceeds the detoxifying and regenerating capacity of the hepatocytes [2], [3]. Hepatotoxicity occurrence is mainly associated with drug consumption [4], [5]. Apart from drugs, genetics, age, lifestyle, and environmental factors enhance the development of hepatotoxicity [6], [7]. Different methods that have been employed for the diagnosis of drug-induced hepatotoxicity have not accurately proven the hepatotoxic effect of drug-drug interactions or drug-herb interactions [8], [9]. Although not specific for DILI, serum levels of alanine aminotransferases (ALT), aspartate aminotransferase (AST), alkaline phosphatase (ALP) and total bilirubin (TB), remain hallmark for detecting and classifying liver damage [10].

Oxidative stress is a primary mechanism leading to hepatic injury [11], [12]. The generation of oxidative stress, mitochondrial dysfunction, and endoplasmic reticulum stress from metabolic activation of chemically active intermediate metabolites that covalently binds macromolecules can cause hepatocyte damage [13]. Recent research revealed that most of the human diseases have a link to oxidative stress and this also increases the focus in identifying antioxidants as part of the solution [14]. During the state of overwhelming cellular ROS production, the antioxidant capacity succumbs, and oxidative stress ensues. ROS has been shown in various studies to activate and repress NfkB, which aided in identifying that NfkB pathway plays a pro- or antioxidant role in the setting of oxidative stress [15]. Even the cholestasis-associated hepatic and renal injury has been successfully improved using antioxidant edaravone [16]. There is also a known interference of phytochemicals with the NFkB pathway which has been shown by curcumin, resveratrol, pterostilbene, punicalagin, macranthoin G, salidroside, 4-O-methylhonokiol, lycopene, genistein, obovatol and gallic acid [17]. These findings shifted a perception that has been created around the safety of natural products.

Though natural products require to be studied for toxicity, there is a huge research output that reports on the beneficial effects of natural products in the liver. Salvoza, et. al, recently reviewed the benefits from the coffee compound of which tied to the synthetic drug development gives hope for future therapeutics against nonalcoholic fatty liver disease (NAFLD) [18]. The Iranian indigenous probiotics also were also recently reported to ameliorate the NAFLD induced reproductive toxicity that is due to oxidative stress [16]. The use of traditional and complementary medicine is gaining support and it is incorporated to the global disease intervention strategy according to the WHO global report on traditional and complementary medicine 2019 [19]. However, herbal and dietary supplements regulations vary across the globe [20]. Rooibos tea is produced from a South African endemic plant *Aspalathus linearis* and has been proven to possess many health benefits [21], [22], [23]. The health benefits of Rooibos tea include anti-oxidant, anti-diabetic, anti-inflammatory [24], [25], hepatoprotective, and chemoprotective effects [26], [27], [28], [29]. Despite the lack of clinical trials on Rooibos there are case studies that associate it with liver toxicity that manifests via elevated platelet count [30]; and elevated liver enzymes [31]. In this study we used a pharmaceutical grade green Rooibos extract (Afriplex ™ GRT) in HepG2/C3A cells and Sprague Dawley rats to assess toxicity of Rooibos.

## 2 Materials and methods

### 2.1 Source and chemical composition of aspalathin-rich green extract

A pharmaceutical grade aspalathin enriched green Rooibos extract (Afriplex™ GRT) was obtained from the manufacturer Afriplex (Pty) Ltd (Paarl, South Africa). The Afriplex™ GRT used in this study contained 12.8% of aspalathin per 100 g of Afriplex™ GRT as reported in the study by Patel et al.*,* [32].

### 2.2 Cytotoxicity testing

#### 2.2.1 Cell line and culture

A sub-clone of HepG2, human hepatocellular carcinoma (HepG2/C3A) [ATCC CRL-10741] cell line was obtained from the American Type Culture Collection (Manassas, Virginia, USA). The cells (1.6 ×10^5^ cells/mL) were cultured in Eagle’s Minimum Essential Medium (EMEM) containing sodium pyruvate and Non-Essential Amino Acids NEAA (Lonza, Houston, Texas, USA), supplemented with 10% Fetal Bovine Serum (FBS) (Highveld Biological, Lyndhurst, South Africa), and 1% of 2 mM L-Glutamine, 1% of penicillin and streptomycin at 37 °C and 5% CO_2_. The media was changed every second day until the cells reached confluency of 80% and cell suspension (22 000 cells) was seeded per well on a 96 well plate.

#### 2.2.2 Mitochondrial activity and membrane integrity

The 100 μg/mL aqueous Afriplex GRT™ working stock was prepared using cell culture grade water (Sigma Aldrich, Missouri, USA). A log dilution of the working stock was used to prepare treatments (1000 µg/mL 100 µg/mL, 10 µg/mL, 1 µg/mL, and 0.1 µg/mL) in 8 mM HEPES-buffered Krebs Ringer Bicarbonate glucose medium. The HepG2/C3A cells were exposed to the extract at different concentrations and incubated for 1 h, 24 h or 48 h. The viability of cells was tested in triplicates using a 96-well clear plate or white opaque microtiter plates on a Bio-Tek ELx800 plate reader (Bio-Tek, Vermont, USA). The adenosine triphosphates (ATP) ViaLight™ plus Kit (Lonza, Pennyslvania, USA) was used to determine ATP content and expressed in percentages of the control. The MTT activity was determined using the (3-(4, 5-Dimethyl-2-thiazolyl) 2, 5-diphenyl-2 H-tetrazolium bromide) dye as detailed in the study by Mosmann, [33].

### 2.3 Sub-chronic toxicity of Afriplex™ GRT in Sprague-Dawley rat model

#### 2.3.1 Animals, diet, housing environment and experimental design

The experimental approach on animals was performed in line with the United States of America Food and Drug Administration immunotoxicity testing guidelines in research (Hinton, n.d). All the protocols used were approved by the University of Zululand Research Ethics Committee (Reg. no. UZREC171110–030) for the use of animals [34]. Nineteen-day-old male (n = 25) and female (24) Sprague-Dawley rats weighing (78.1 ± 17.8 g) were obtained from the University of Zululand Department of Biochemistry and Microbiology animal facility. The rats were body weight matched and assigned to five groups caged in two or three rats of the same gender per cage. The rats were kept on a corn cob bedding and received water and standard rat chow (6% simple sugars, 5.9% fats, 44% polysaccharides, 27% protein (w/w), energy 3.02 kcal/g) ad *libitum*. The environmental conditions were kept at 23–25 °C, relative humidity 50%, 12 h light and dark cycle. The rats were divided into five groups of 10 rats/group (n = 5 males and n = 5 females) except for the control group that consisted of (n = 5 males and n = 4 females) and the sample sizes were derived from a previous published study by van Der Merwe and co-worker, (2015) [35] with modification and considerations from the toxicity study guidelines [36], [37].

#### 2.3.2 Afriplex™ GRT administration and induction of liver toxicity

The adjusted dose of Afriplex™ GRT used for this study was based on human consumption of Rooibos tea and the amount beyond human consumption was included to establish toxic amount. This was in consideration of the dose that was established for monkey and converted using Reagan-Shaw and Ahmad approach [38]. The treatments were administered in jelly (Moirs, South Africa) cube prepared as detailed in Layman, et. al., 2019 [39]. The groups were subdivided to Control (jelly cube without Afriplex™ GRT); 10 mg/kg, 100 mg/kg, 300 mg/kg (given jelly cube with Afriplex™ GRT 10, 100 or 300 mg/kg, respectively); and CCl4 (jelly cube without Afriplex™ GRT and injection with 0.8 mg/kg CCl4 in a 1:1 ratio with olive oil 24 hrs prior to termination). The duration of the experiment was 90 days, and the treatment was administered daily at 8 am.

#### 2.3.3 Sample collection

The rats were fasted overnight prior to termination. On the day of termination 15 mg/kg of sodium pentobarbital was injected intraperitoneally. An incision was performed, and the rats were killed by exsanguination. Blood samples were collected in BD vacutainer blood collection tubes (Becton Dickinson Pty Ltd, Johannesburg, South Africa) for clinical biochemistry analysis; and the livers were collected, washed in saline solution, weighed, and fixed in 10% formalin for histology. Parts of the liver were snap frozen and stored at − 80 °C for further analysis.

#### 2.3.4 Clinical chemistry parameters and liver histopathology examination

Liver enzymes ALT, AST, and ALP were measured from the serum. Other serum measured parameters include total protein and albumin as well as total bilirubin, urea, uric acid, and creatinine that were measured by the specialist at Lancet pathology laboratories (Empangeni, KwaZulu Natal, SA) according to their procedure.

The liver samples fixed in 10% formalin (Merck-Millipore, Billerica, United States) were assessed for histopathological changes. The tissues embedded into paraffin-wax blocks were cut into slices not thicker than 5 µm and transferred into slides. The slide-embedded specimens were stained with hematoxylin and eosin (H&E) stain (Merck-Millipore) to evaluate steatosis and inflammation. To assess the response to oxidative stress the immunohistochemistry of glutathione s-transferase pi (GST-pi) was performed. Microscopy image capturing done on tissue sections using Nikon eclipse Ti microscope (Advanced Laboratory Solutions, South Africa) with inverted camera and representative images were selected for each group. The images were analyzed by Image J software version j 1.52r (US National Institutes of Health, Bethesda, United States) to study the difference in % area occupied by fat to that stained with H&E. The histological features were described and reported as mild, moderate, and severe. The non-alcoholic fatty liver disease activity score was used to estimate the severity of steatosis. The grading score used ranged from 0 to 3, 0 signifying absence of steatosis or steatosis < 5%, 1 presenting steatosis 5%− 33%, 2 defined as occurrence of steatosis of 33%− 66% and 3rd grade for steatosis > 66% [40].

#### 2.3.5 Western blotting

The samples that were previously frozen were used for immunoblotting. The tissues of 96–110 mg were weighed and washed in cold phosphate buffered saline (PBS) (Merck-Millipore) in a 2 mL tube. The PBS was replaced with 500 µL of cold lysis buffer, placed in a precooled tissue lyser blocks and a steel ball was added to the tubes. The samples were homogenized in a tissue lyser (Qiagen, USA) at 25 Hertz for 60 s, cooled in ice for 60 s, this was repeated 5 times. The samples were then centrifuged at 13000 rpm for 15 min at 4 °C. The supernatant was collected for further analysis. The protein concentration of all the liver homogenates were estimated in DC protein assay (Bio Rad, Hercules) and bovine serum albumin (Bio Rad, Hercules) was used as a standard. Extracted protein, 30 µg protein per well were loaded on a 12% SDS-polyacrylamide gel electrophoresis (SDS-PAGE), followed by a semi-dry transfer of the SDS-PAGE into polyvinylidene fluoride (Bio Rad, Hercules, USA) membranes. The membranes were Ponceau stained for protein transfer validation. The PVDF membranes were blocked for 90 min TBST that contains 5% non-fat milk on a shaker. After blocking, the membranes were probed with the primary antibody (NF-kB (1:2000), Bax (1:1000), Caspase 3 (1:1000), CYP3A4 (1:1000) and Bcl-2 (1:1000) in 1x Tris buffered saline with 0.1% Tween 20 (TBST) and incubated overnight on a shaker at 4 °C. Thereafter, the membranes were washed three times for 10 min in a TBST at room temperature and incubated with the secondary antibody in 2.5% non-fat milk in TBST for 90 min. The membranes were washed and ECL substrate kit was used for detection of protein bands. The results were normalized to the house-keeping gene β-Actin (1: 1000).

### 2.4 Statistical analysis

GraphPad Prism version (5.04) software was used to analyze the data. One-way analysis of variance (ANOVA) was used to compare the data. The data were presented as mean ± SEM. The Dunnett’s *post-hoc* test was used to test the significance and the Bonferroni *post-hoc* multiple comparison test was employed for comparison amongst the treatment groups. The difference of *p-value*< 0.05 was statistically significant.

## 3 Results

### 3.1 The effect of Afriplex™ GRT on HepG2/C3A cell viability

**Fig. 1:**
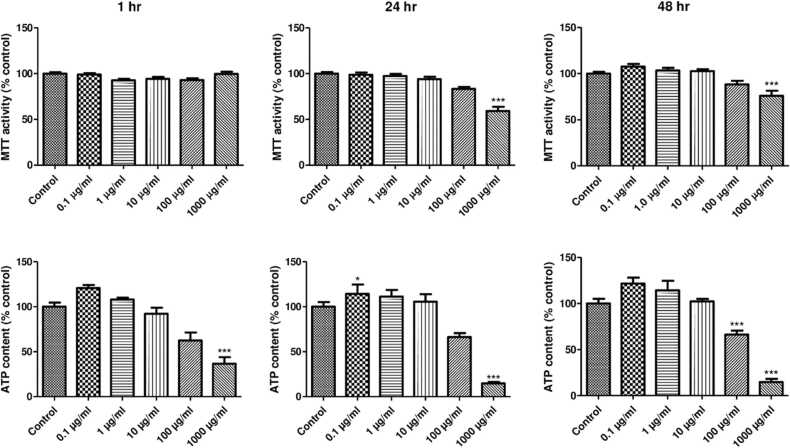
The effect of Afriplex™ GRT on cell viability on HepG2/C3A cells using MTT and ATP assays. The effect of Afriplex™ GRT on HepG2/C3A cells viability after 1 h (A), 24 h (B), and 48 h (C) incubation using MTT assay. Cell viability was confirmed by ATP assay after 1 h (D), 24 h (E), and 48 h (F) incubation. The data is presented as mean ± SEM of three independent experiments, * ** p-value < 0.001 versus control, *p < 0.05.

The Afriplex™ GRT treatment reduced cell viability for the HepG2/C3A treated with 1000 µg/mL at 24 and 48 hrs. (Fig. 1). MTT assay showed that Afriplex™ GRT treatment (1000 µg/mL) significantly reduced cell viability by ≥ 50% after 24 h incubation compared to the control. The confirmation of the similar experiments using ATP assay showed that Afriplex™ GRT at 1000 µg/mL reduce cells viability by ≥ 80%.

### 3.2 Sub-chronic Sprague Dawley rat toxicity study

**Fig. 2:**
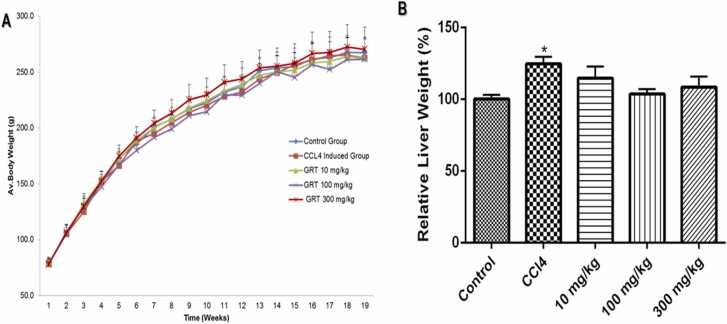
The effect of Afriplex™ GRT on body weight (A) and relative liver weight (B) of Sprague-Dawley rats. The body weights were monitored weekly and recorded as mean ± SEM and relative liver weight recorded at termination and calculated by dividing the body weight of the rat with the liver weight expressed relative to the control set at 100%, presented as Mean ± SEM. (n = 10 rats/group).

The Afriplex™ GRT had no significant effect on body weights or relative liver weights **(**Fig. 2). There were no significant changes in the food and water intake after 90 days when compared to the control. Neither were any adverse clinical signs or mortalities encountered during the period of the study.

### 3.3 Serum biochemical parameters

**Fig. 3:**
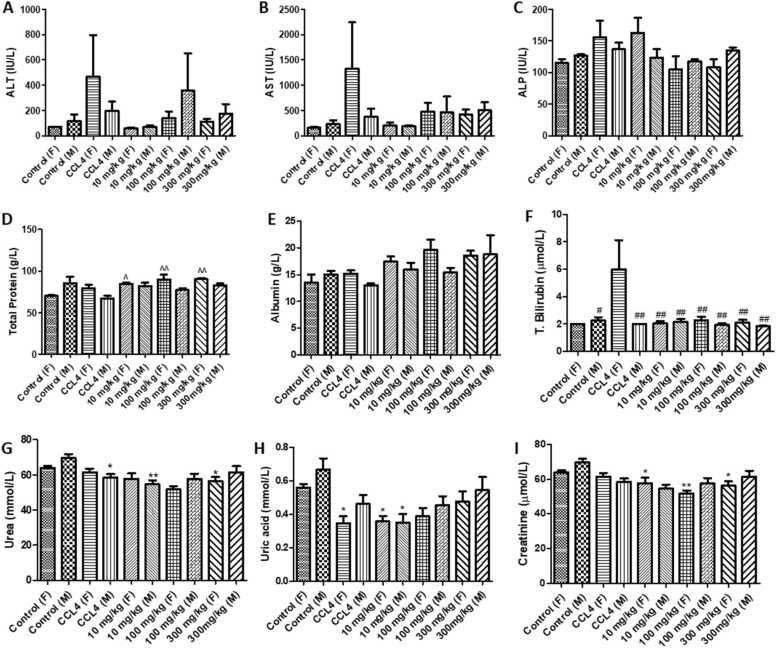
Liver function ALT (A), AST (B), ALP (C), total protein (D), albumin (E), total bilirubin (F), urea (G), uric acid (H) and creatinine (I) levels for the untreated control group and treated with CCl_4_ administered 24 h before terminations (0.8 mg/kg; positive control) and Afriplex™ GRT administered for 90 days (10, 100, 300 mg/kg) treated groups (n = 10 rats/group). Results represent the mean ± SEM. Statistical analysis One-way ANOVA, *p < 0.05 versus the vehicle control group (M), * *p < 0.01 versus vehicle control (M), #p < 0.05 versus CCl_4_ (F), ##p < 0.01 versus CCl_4_ (F), ^p < 0.05 versus CCl_4_ (M) and ^^p < 0.01 versus CCl_4_ (M).

Serum biochemistry results (Fig. 3) showed that CCl_4_ administered 24 h before terminations had no significant effects in liver enzymes ALT, AST, and ALP (Fig. 3 **A-C**). However, the CCl_4_ elevated these enzymes more in female compared to their male counterparts. Exposing male rats to CCl_4_ reduced the serum total protein which was significant compared to the levels in the females treated with Afriplex™ GRT (Fig. 3**D**)**.** A similar trend to that of total protein was also observed with serum albumin in males treated with CCl_4_ but the results were not significant (Fig. 3**E**). Serum bilirubin for the females that were injected with CCl_4_ were significantly high compared to all the groups (Fig. 3**F**). The serum urea levels dropped significantly for the male rats on CCl_4_ and 10 mg/kg Afriplex™ GRT (Fig. 3**G**). Females injected with CCl_4_ had significantly reduced serum urea levels compared to controls. This significant reduction in urea levels were also observed for both males and females on 10 mg/kg Afriplex™ GRT compared to male controls (Fig. 3**H**). A. All dosages of Afriplex™ GRT significantly reduced levels of serum creatinine in female rats compared to control (Fig. 3**I)**.(Table 1).

**Table 1:** Steatosis severity in Sprague Dawley rats in different treatments.

	*Control*	*CCl*_*4*_	*Afriplex*^*TM*^*GRT (10 mg/mL)*	*Afriplex*^*TM*^*GRT (100 mg/mL)*	*Afriplex*^*TM*^*GRT (300 mg/mL)*
*% steatosis*	≤ 5%	≤ 15%	≤ 10%	≤ 10%	≥ 10%
*Micro*	+	> ++ +	+ +	+ +	+ ++
*Medio*	+ +	+ ++	+ +	+ +	+ +
*Macro*	0	+ ++	+	+	+ +

Notes: Microvesicular steatosis is characterized by small intracytoplasmic fat vacuoles (liposomes) which accumulate within hepatocytes.

Mediovesicular steatosis is characterized by a mixture of small and large fat droplets

Macrovesicular steatosis is characterized by the fat vacuole that completely occupying the cytoplasm with the nucleus completely replaced

### 3.4 Histopathology

**Fig. 4:**
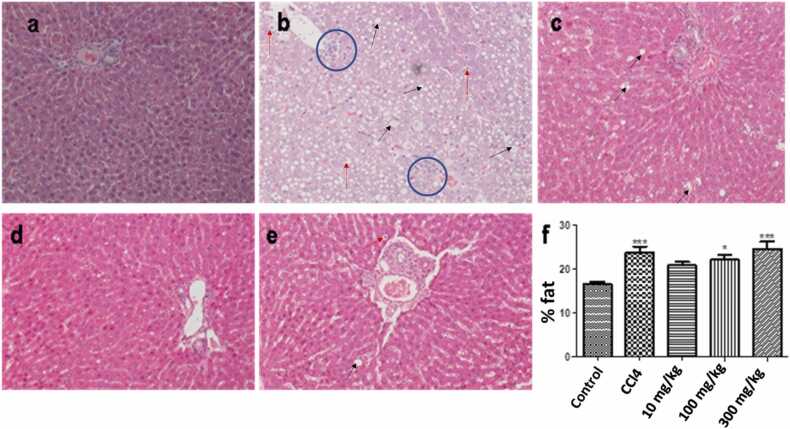
*Representative liver hematoxylin and eosin (H&E) sections from different treatment groups.* Normal liver tissue demonstrating typical liver morphology with hepatocytes and sinusoids (A); CCl_4_ treated positive control group (B) demonstrates severe diffuse hepatocellular micro- and macro-vesicular degeneration (black arrows) with pyknotic nuclei (red arrows) showed central vein without micro- and macro-vesicular degeneration; The 10 mg/kg treated group (C); The 100 mg/kg treated group (D) showing minor hepatocellular micro-vesicular degeneration changes present; The 300 mg/kg treated group (E) shows a focal area of hepatocellular micro-vesicular degeneration with more acinar spaces instead of macro-vesicular degeneration. H & E 200 x magnification.

**Fig. 5:**
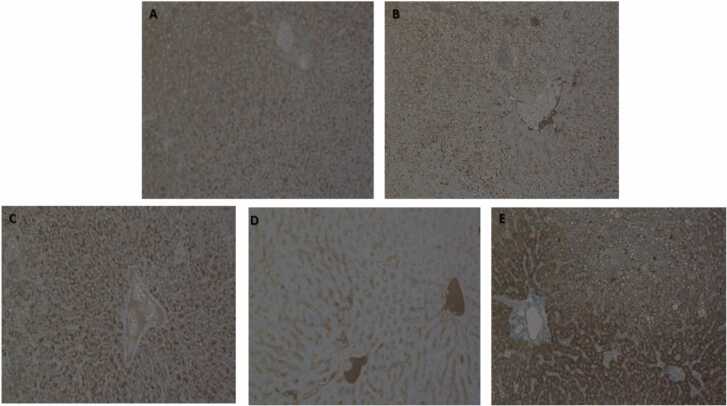
*Representative liver Glutathione S-Transferase (GST-Pi) sections from different treatment groups*. The vehicle untreated group (A). The 10 mg/kg treated group (B). The 100 mg/kg treated group (C). The 300 mg/kg treated group (D). CCl_4_ treated positive control group (E). GST-Pi x 200 magnification.

The histopathological features of steatosis were classified based on changes in the morphological features. Microvesicular and macrovesicular steatosis denoted the presence of small innumerable lipid droplets with no deformation of the hepatocyte nucleus and large lipid droplets displacing the nucleus, respectively [41]. Liver histopathology results demonstrated mild micro-and-macrovesicular steatosis in females and males of the control group. In comparison to the control, mild to moderate micro-and-macrovesicular steatosis was observed in the groups treated with Afriplex™ GRT (100, and 300 mg/kg). CCl_4_ treated group (positive control) displayed diffused marked hepatocellular damage and extensive micro-and macrovesicular degeneration of hepatocyte with pyknotic (apoptotic) nuclei (Fig. 4).(Fig. 5).

### 3.5 Effect of Afriplex™ GRT on protein expression

**Fig. 6:**
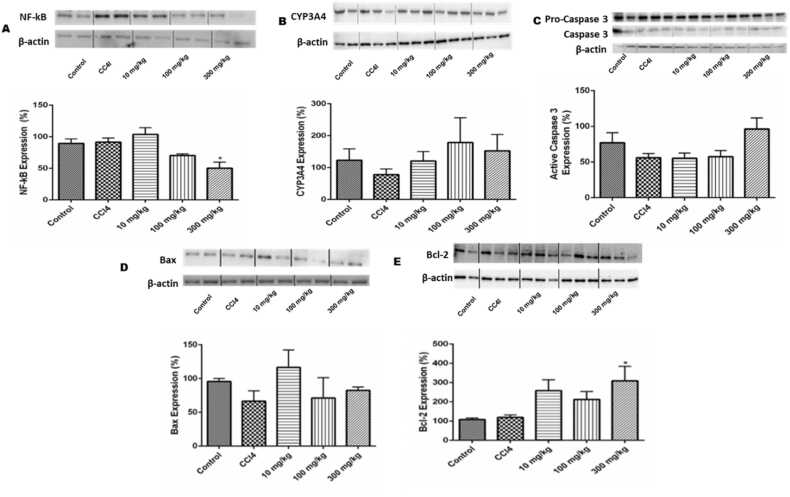
Relative expression of nuclear factor-kB (A), CYP3A4 (B), Caspase 3 (C) and Bax (D) and Bcl-2 (E)assessed by Western blot in liver of male rats (n = 5 rats /group), NF-kB p65, Bax, Caspase 3, CYP3A4 and Bcl-2 primary antibodies were used on liver samples of untreated, treated with CCl4 (0.8 mg/kg; positive control) 24 h before termination or Afriplex™ GRT (10, 100, 300 mg/kg) administered for 90 days. Results represent the mean ± SEM in three independent experiments relative to the house-keeping gene, B-Actin. *p < 0.05 versus the control group.

Treatment with Afriplex™ GRT (10 mg/kg and 100 mg/kg) did not have any significant effect on the protein expression of NF-kB, Bax, caspase 3, and CYP 3A4, whilst Afriplex™ GRT (300 mg/kg) significantly (p < 0.05) reduced the expression of NF-kB but had no effect on the expression of Bax, caspase 3 and CYP3A4 (Fig. 6).

## 4 Discussion

In the present study HepG2/C3A cells and Sprague Dawley rats were used to test the hepatotoxic effect of pharmaceutical grade aspalathin rich green Rooibos (Afriplex™ GRT). The confluent HepG2/C3A cells were subjected to different concentrations of Afriplex™ GRT, followed by assays that measure the severity of exposure to the treatment. The Sprague Dawley rats were given maintenance diet and treated with different sub-chronic (90 days) dosages of Afriplex™ GRT. As a reference an acute dose of CCl_4_ (0.8 mg/kg) was injected intraperitoneally to induce hepatotoxicity 24 hrs before termination and the Afriplex™ GRT treated groups and the control group were injected with olive oil. Serum biochemistry was analyzed, and the livers were subjected to qRT-PCR and Western blot to compare gene and protein expression.

The outcome of our study showed that the Afriplex™ GRT can reduce cell viability at a concentration of 1000 µg/mL at 24 and 48 hrs incubation. In line with what has been reported before about the interference of some phytochemicals for certain assays, MTT did not show the reduction in cell viability that was as prominent as that seen with the ATP assay. The study on epigallocatechin gallate a polyphenol in green tea showed two-fold IC50 using MTT, and MTS compared to dyes that are quantifying ATP and DNA [42]. This interference of polyphenols was observed in our study as well where ATP showed ∼10% viability while MTT showed the viability of ∼70% after 48 hrs in the incubation with the highest concentration (1000 µg/mL) despite the huge patches that were seen under the microscope. From literature it has been suggested that normalizing the MTT assay to polyphenol tested aids to normalize the color effect that the polyphenols add [43]. For this current study, this correction was not performed.

Males and females in different groups did not show significant differences in serum liver enzymes. However, the levels of serum liver enzymes for CCl_4_ injected females were high compared to that of their male counterparts. The differences in response to CCl_4_ have been previously reported and show that female rats respond more severely than males to acute CCl_4_ [44]. Expectedly CCl_4_ due enzymatic activation releases CCl_3_ free radical which in turn disrupts the structure and function of lipid and protein macromolecule in the membrane of cell organelles leading to increased liver enzymes and bilirubin [45]. Treatment with Afriplex™ GRT increased serum total proteins in female rats than in males. Using CCl_4_ in males led to the reduction in serum total protein. Female rats also experienced significantly increased levels of bilirubin because of CCl_4_ treatment which is in line with the findings by Al-Yahya et al. [46]. This rise in bilirubin levels did not happen in males on CCl_4_. This may be in the differences gender in response to CCl_4_ mentioned above. Overall, there was no indication of toxicity posed by Afriplex™ GRT in reference to what was observed with CCl_4_. In a similar study to the current study, Van de Merwe et al. [35] shows that green rooibos extract has the potential to increase liver enzymes. Whilst in their study, using a very high dose of the extract (1900 mg/kg BW) demonstrated that ALP was significantly increased but not AST or ALT. Looking into the results of both studies it appears that dose determines either hepatocellular predominant enzyme pattern (elevated ALT) with increased AST: ALT ratio (2.26 vs. 3.7) as is seen in this study or a cholestatic predominant pattern (increased ALP) described by Van der Merwe [35].

Severe accumulation of lipid vacuoles within the hepatocyte usually seen in experimental models has been associated with increased oxidative stress, increased hepatic enzymes, activation of NF-kB, and increased expression of pro-inflammatory cytokines and TNF-α, thereby exacerbating hepatocellular damage [47]. The accumulation of fatty acid which was observed to be severe in CCl_4_ treated group can be interpreted as vesicular steatosis [48]. Increased hepatic steatosis contributes to the development of insulin resistance and is correlated with obesity [49]. In our study the livers of the CCl_4_ showed the steatosis more than all the groups. The Afriplex™ GRT showed to influence the acinar spaces size as seen at 300 mg/kg dose. The CCl_4_ also showed positive reaction to GST-Pi which is a marker of toxicity as it is getting more expressed when toxicity is experienced because it plays a role in xenobiotic metabolism. This was not the case in other groups.

Metabolism in the liver involves the activity of the cytochrome P_450_ enzymes. Although the limit in the bioavailability of aspalathin still exists, a study documented that GRT undergoes liver metabolism and is excreted in urine [50]. Interactions of Rooibos and drugs have been discovered to either inhibit or enhance the activation of genes and proteins [51]. Proteins are critical elements in toxicity studies such that their activation provides insight regarding pathways involved. For instance, the release of cytochrome c which occurs because of disruption of mitochondrial membrane plays a significant role in the activation of caspase 3, a member of the apoptotic signaling pathway [52]. The activation of caspase 3 is prevented by the overexpression of Bcl-2, an important anti-apoptotic protein including Bax [53]. Using antibodies that investigate the apoptotic signaling pathway, it was discovered that GRT has no significant effect on the expression of caspase 3 and Bax. Interestingly, the protein expression of NF-kB was significantly reduced by the 300 mg/kg BW GRT treatment, suggesting that the reduction of NF-kB could be a functional mechanism of preventing cell death and improving cell viability after GRT treatment [54]. Studies have shown that the inhibition of tumor promoter proteins such as NF-kB is chemopreventive and that medicinal plants consisting of polyphenols have high inhibition factor [55], [56]. The expression of NFkB in our study for the Afriplex™ GRT treated rats showed a dose dependent reduction. This is an interesting finding because there are studies that report on the benefits of inhibiting NFkB pathways as a strategy to blunt bile acid-induced hepatic and renal toxicity [57], [58]. More need to be explored with regards to Afriplex™ GRT and the possible mechanism it has on the NFkB pathway for future studies.

## 5 Conclusion

The findings of our study suggest that Afriplex™ GRT has a dose-dependent hepatotoxic effect on HepG2/C3A. There were no correlating findings that show the toxicity in Sprague-Dawley rat. These findings prove that regardless of the general perception that herbal supplements are safer than conventional drugs, undesirable responses may still be observed especially with products enriched with bioactive compounds as seen on the cell work. It is worth noting that Afriplex™ GRT (90-days daily dose) induced minimal adverse effect compared to the CCl_4_ (24-hours response) treated group which was used as a reference for toxicity. Our findings show that Afriplex™ GRT is having a beneficial effect that is through its action on NFkB pathway but more need to be done to elucidate how this mechanism works.

## CRediT authorship contribution statement

Ntandoyenkosi and Kwazi wrote the paper. The supervision of the project was by Christo Muller, Paul Kappo, Kwazi Gabuza and Rebamang Mosa. Johan Louw contributed to the resources that were required for the project. Thendo Mabuda contributed in the analysis of histological images and writing of the paper. All the authors contributed to the writing and editing of the paper.

## Declaration of Competing Interest

The authors declare that they have no known competing financial interests or personal relationships that could have appeared to influence the work reported in this paper.

## Data Availability

Data will be made available on request.
